# Morbidly obese parturient: Challenges for the anaesthesiologist, including managing the difficult airway in obstetrics. What is new?

**DOI:** 10.4103/0019-5049.72639

**Published:** 2010

**Authors:** Durga Prasada Rao, Venkateswara A Rao

**Affiliations:** Department of Anaesthesiology, Siddhartha Medical College, Government General Hospital, Government of Andhra Pradesh, Vijayawada, India

**Keywords:** Adipocyte, complications, difficult airway, morbidly obese, parturient, regional, team work

## Abstract

The purpose of this article is to review the fundamental aspects of obesity, pregnancy and a combination of both. The scientific aim is to understand the physiological changes, pathological clinical presentations and application of technical skills and pharmacological knowledge on this unique clinical condition. The goal of this presentation is to define the difficult airway, highlight the main reasons for difficult or failed intubation and propose a practical approach to management Throughout the review, an important component is the necessity for team work between the anaesthesiologist and the obstetrician. Certain protocols are recommended to meet the anaesthetic challenges and finally concluding with “what is new?” in obstetric anaesthesia.

## INTRODUCTION

“You can tell the condition of a nation by looking at the status of its women”

*Jawaharlal Nehru*

With technological advances, the world is moving fast and, at the same time, the population worldwide is becoming fatter and fatter. Obesity now prevails in all sections of people irrespective of whether it is a developed or developing or a poor country. As the person becomes rich in his fat the economic reserves of that country becomes thin. The World Health Organization (WHO) predicts that there will be 2.3 billion overweight adults in the world by 2015, and more than 700 million of them will be obese.[1]

Obesity is associated with more than 30 medical conditions, including diabetes, high blood pressure, high cholesterol and triglycerides, coronary artery disease (CAD), sleep apnoea, strokes, gallbladder disease and cancers of the breast and colon.[2]

## DEFINITION OF OBESITY

Obesity is a disorder of energy balance. It is derived from the Latin word obesus, which means fattened by eating. Obesity is the state of excess adipose tissue mass. The most widely used method to gauge obesity is body mass index (BMI), which is equal to weight/height^2^ (kg/m^2^), also known as Quetelet’s Index [Table 1].

**Table 1:** Classification of weight status and risk of disease

	BMI (kg/m^2^)	Obesity class	Risk of disease
Underweight	<18.5		
Healthy weight	18.5–24.9		
Overweight	25–29.9		Increased
Obesity	30–34.9	Class I	High
Obesity	35–39.9	Class II	Very high
Extreme obesity	>40	Class III	Extremely high

More recently, the categories of super morbid obesity, >50 kg/m^2^, and ultra obesity, >70 kg/m^2^, have been recognized. The American College of Obstetrics and Gynecology recommends height and weight measured at the first prenatal visit to calculate the BMI.[3]

## PREVALENCE

America is the fattest country in the world. The poor in the developed and the effluent in the developing countries are obese. Data from the national health and nutrition examination surveys show that the percentage of the American adult population with obesity BMI more than 30 has increased from 14.5% to 30.5% by 2000. As many as 64% of the US adults >20 years of age were overweight between the years of 1999 and 2000. The extreme obesity BMI >40 has also increased and effects 4.7% of the population. In 2003–4, 17.1% of the US children and adolescents were overweight and 32.2% of the adults were obese.[4,5] In the United Kingdom, there were 665 women with extreme obesity in an estimated 764,387 women delivering, representing an estimated prevalence of 8.7 cases per 10,000 deliveries (95% CI 8.1–9.4).[6]

## PREVALENCE IN INDIA

In India, obesity has reached an epidemic proportion, affecting 5% of the country’s population.[7] The obesity trend has been found to be higher in women of Jalandhar District (Punjab) as compared with other Indian women populations studied so far, except the women population of West Bengal (Das and Bose 2006) and the Punjabi Bhatia women of Jaipur.[8–10] Indians are genetically susceptible to weight accumulation, especially around the waist.

Saha and others[11–13] submitted to the National Family Health Survey that there is an increased trend towards overweight or obesity in Indian women from 10 in 1998–9 to 14.6 in 2005.

In India, states that topped the list of rates of obesity were Punjab (30.3% males, 37.5% females), Kerala (24.3% males, 34% females) and Goa (20.8% males, 27% females).[14]

## ADIPOCYTE

**Figure 1:**
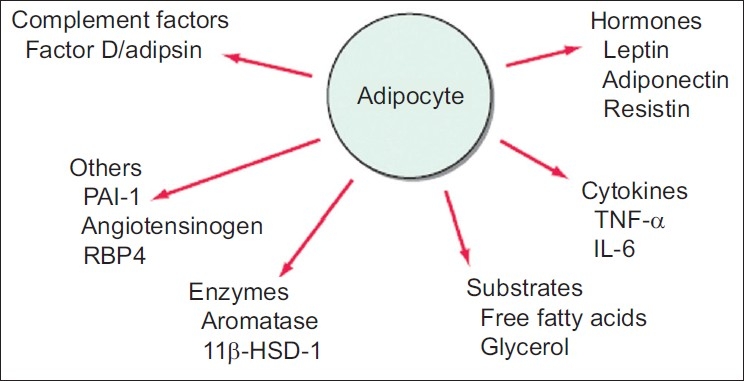
Factors released by the adipocyte that can affect peripheral tissues. PAI, plasminogen activator inhibitor; TNF, tumour necrosis factor; RBP4, retinal binding protein 4. (From Harrison’s Principles of Internal Medicine, 17^th^ edition)

Although adipocyte [Figure 1] has generally been regarded as a storage depot for fat, it is also an endocrine cell that releases numerous molecules. These include energy balance regulating hormone leptin, cytokinin such as tumour necrosis factor alpha and interleukin (IL)-6, compliment factors such as factor-D, prothrombotic factors such as plasminogen activator inhibitor 1 and a component of the blood pressure regulating system, angiotensinogen. These factors play a role in the physiology of lipid homeostasis, insulin sensitivity blood pressure control, coagulation and vascular health, and are likely to contribute to obesity-related pathologies.

## ROLE OF GENES VERSUS ENVIRONMENT

Obesity is commonly seen in families and heritability of body weight is similar to that for height. Inheritance is not Mendelian. Whatever the role of the genes, it is clear that the environment plays a key role in obesity. However, identification of ob gene mutation in genetically obese ob/ob mice represented a major breakthrough in this field. The OB gene is present in humans and is expressed in fat.

## PHYSIOLOGICAL CHANGES IN PREGNANCY AND IN THE RESPIRATORY SYSTEM OF MORBIDLY OBESE PREGNANT PATIENTS

Changes in the respiratory system during pregnancy are manifest as alterations in the upper airway, minute ventilation, lung volumes and arterial oxygenation [Table 2].

**Table 2:** Respiratory changes in pregnancy obesity and combined

Parameter	Pregnancy	Obesity	Combined
Progesterone level	↑	↔	↑
Sensitivity to CO_2_	↑	↓	↑
Tidal volume	↑	↓	↑
Respiratory rate	↑	↑↔	↑
Minute volume	↑	↓↔	↑
Inspiratory capacity	↑	↓	↑
Inspiratory reserve volume	↑	↓	↑
Expiratory reserve volume	↓	↓↓	↓
Residual volume	↓	↓↔	↑
Functional residual capacity	↓↓	↓↓↓	↓↓
Vital capacity	↔	↓	↓
FEV_1_	↔	↓↔	↔
FEV_1_/VC	↔	↔	↔
Total lung capacity	↓	↓↓	↓
Compliance	↔	↓↓	↓
Work of breathing	↑	↑↑	↑
Resistance	↓	↑	↓
V/Q	↑	↑	↑↑
PaO_2_	↓	↓↓	↓
PaCO_2_	↓	↑	↓

Respiratory changes in pregnancy, obesity and combined, Anaesthesia 2006; 61; 36-48; adopted from Sarvanankumar *et al*., Obesity and obstetric anaesthesia.

### Minute ventilation

**Figure 2:**
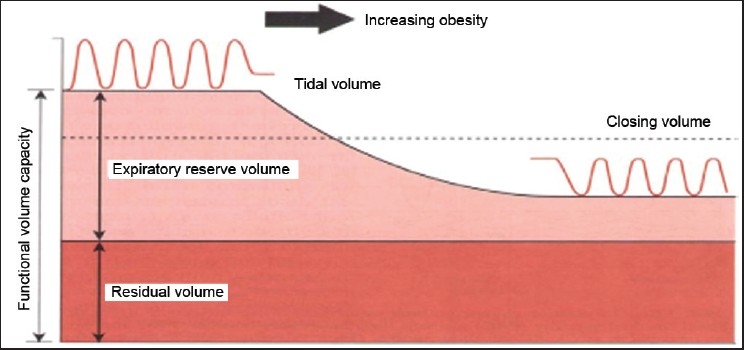
Effect of obesity on lung volumes and closing pressure (Errol Lobo)

An increase in minute ventilation is one of the earliest and most dramatic changes in the respiratory function during pregnancy, whereby chest wall compliance is decreased. As a result, the work of breathing is increased and ventilation become diaphragmatic and position-dependent. Pulmonary function studies in obesity suggest a restrictive pattern of lung disease, and the most constant changes are reduction in expiratory reserve volume, vital capacity and functional residual capacity; however, the inspiratory capacity is increased in obese parturients. Increased closing volume with decreased expiratory reserve volume results in underventilation of the dependent lung regions [Figure 2].

Obstructive sleep apnoea is not uncommon in obese women who become pregnant. Pregnancy has some protective effects on sleep apnoea despite the hyperemia of nasal passages.

The combination of increased minute ventilation and decreased functional residual capacity (FRC) demonstrate the rate at which changes in the alveolar concentration of an inhaled anaesthetic drug can be achieved. Induction and emergence and depth of anaesthesia are notably faster.

Ventillatory changes are more important than circulatory alterations in determining the alveolar concentration of inhaled anaesthetics. Dose requirements for volatile anaesthetics drugs are reduced during pregnancy. Thus, lower concentrations of inhaled anaesthetics may result in a loss of protective upper airway reflexes during the delivery of inspired concentration of anaesthetics that are usually considered safe.

## CARDIOVASCULAR CHANGES

**Figure 3:**
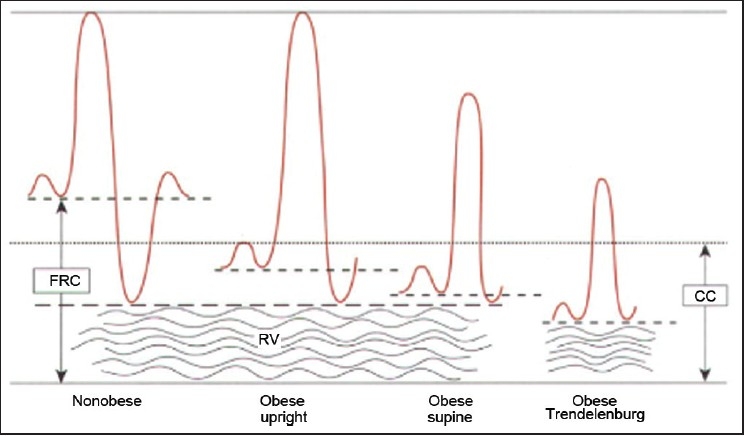
Effect of positioning on the morbidly obese patients (Errol Lobo)

An increased demand for oxygen of the obese individuals results in an increased workload on the heart. The cardiac output and blood volume are increased. Each kilogram of fat contains 3,000 m of blood vessels. Other important problems are pulmonary arterial hypertension (PAH), left ventricular (LV) hypertrophy, decreased LV contractility, supine hypotension syndrome, ECG changes shows LV strain, flat T waves and low-voltage QRS complex [Table 3]. Different positions of a obese patient has effects on the lung volumes [Figure 3].

**Table 3:** Cardio-vascular changes

Parameter	Pregnancy	Obesity	Combined
Heart rate	↑	↑↑	↑
Stroke volume	↑↑	↑	↑
Cardiac output	↑↑	↑↑	↑↑↑
Blood volume	↑↑	↑	↑
Mean arterial pressure	↑	↑↑	↑↑
Systolic function	↔	↔↓	↔↓
Diastolic function	↔	↓	↓
Systemic vascular resistance	↓		↓
CVP	↔	↑	↑↑
Pulmonary hypertension	Absent	May be present	May be present
Pre-eclampsia			↑↑

Cardiovascular changes in pregnancy, obesity and combined, Anaesthesia 2006;61:36-48; adopted from Saravanankumar *et al*., Obesity and obstetric anaesthesia

## GASTROINTESTINAL CHANGES

Progesterone relaxes the smooth muscles. Consequently, it impairs oesophageal and intestinal motility during pregnancy. Although it was always accepted that the gastric emptying was delayed during pregnancy, it has recently been suggested that gastric emptying is not always delayed in pregnant woman.[15–17]

## OBESITY-ASSOCIATED SYSTEM-WISE COMORBID CONDITIONS

The following conditions are associated with obesity [Table 4].

**Table 4:** Obesity-associated system-wise comorbid conditions

System	Clinical manifestation
Respiratory system	Dyspnoea
	Obstructive sleep apnoea
	Hypoventilation syndrome
Cardiovascular system	Hypertension of pregnancy pre-eclampsia
	Congestive heart failure
	Thromboembolic manifestations
	Pulmonary embolism
Musculoskeletal	Lowback pain
	Immobility
	Osteoarthritic knees and hips
Gastrointestinal	Gastroesophageal reflux, fatty liver, cholelithiasis, hernias, cancer
Endocrine	Type 2 diabetes
	Dyslipidemia
	Polycystic ovarian syndrome

Understanding the nature of involvement of the various systems helps in planning the management of obese parturients.

## MATERNAL COMPLICATIONS

### Hypertension

In a study of 4,100 deliveries in California, the prevalence of pregnancy-induced hypertension was 4.2% in normal weight women and increased to 9.1% in the obese women, the values being 1.2 and 5.3% for what the authors called hypertension.[18] The incidence of gestational hypertension increased from 4.8% in the normal weight group to 10.2% in the obese group (*n* = 1,473) and 12.3% in the morbidly obese group (*n* = 877).[19]

### Gestational diabetes

In a study of 16,102 women, the incidence of Gestational diabetes (GDM) was 2.3% in the control group and increased to 6.3% in the obese group (OR 2.6) and 9.5% in the morbidly obese group (OR 4.0).[19] In a UK study, women with a BMI greater than 30 kg/m^2^ are 3.6-times more likely to develop GDM compared with women with a normal BMI.[2] Diabetes is associated with increasing overweight and obesity. Sixty percent of women have an unplanned pregnancy and may have undiagnosed diabetes. The pregnancy is at increased risk of foetal malformation in addition to foetal macrosomia.

### Maternal deaths

18^th^ in a series of reports within the Confidential Enquiries into Maternal and Child Health (CEMACH) in the UK, in the years 2003–5, there were six women who died from problems directly related to anaesthesia, which is the same as the reported deaths in the 2000–2 triennium. Obesity was a factor in four of these women who died, indicating the magnitude of the problem.[20]

## FOETAL COMPLICATIONS

### Congenital malformations

One case–control study found that women with a BMI greater than 31 kg)/m^2^ had a significantly increased risk of delivering infants with neural tube defect (NTD) and defects of the central nervous system. The increased risk of NTD in infants of obese women was thought to be related to the lower levels of folic acid that reach the embryo due to poor absorption and higher metabolic demands.

### Macrosomia

Several studies have shown that maternal obesity and excessive weight gain during pregnancy are associated with macrosomic babies.[19,21] Obesity and pre-GDM are independently associated with an increased risk of large-for-gestational-age infants, and this impact of abnormal body habitus on birthweight increases with increasing BMI and is associated with significant obstetric morbidity.[22,23]

### Thromboembolism

The risk of thromboembolism is increased in obese parturients. Edwards and others[24] reported 683 obese women (BMI > 29 kg/m^2^) who were matched to 660 women of normal weight (BMI 19.8–26.0 kg/m^2^). The incidence of thromboembolism was 2.5% in the obese women and only 0.6% in the control subjects.[24] The Royal College of Obstetricians and Gynaecologists (RCOG) in the United Kingdom recommends thromboprophylaxis for 3–5 days using low-molecular weight heparin after vaginal delivery for women who are over the age of 35 years and have a pre-pregnancy or early pregnancy BMI >30 kg/m^2^ or weight >90 kg.[25] In addition, the RCOG recommends thromboprophylaxis before and for 3–5 days following caesarean section for women with a pre-pregnancy or early pregnancy BMI >30 kg/m^2^ or with a current weight >80 kg. The RCOG also recommends considering thromboprophylaxis in “extremely obese” women who are hospitalized antenatally.[25,26]

## PRE-OPERATIVE ISSUES

A thorough pre-operative evaluation is essential to minimize surgical complications. All severely obese patients should undergo pre-operative chest radiograph, electro cardiogram (EKG) and laboratory screening with a complete blood count, liver function tests, electrolyte panel, coagulation profile and urine analysis. A pre-operative cardiology consult is highly recommended. Patients with severe obesity and no further cardiac risk factors should be placed on a peri-operative beta blocker, whereas those with identifiable cardiac risk factors should undergo additional non-invasive cardiac testing pre-operatively.

The pre-operative anaesthesia consult should include an assessment of airway and potential vascular access sites. If there is any evidence of pulmonary dysfunction, an arterial blood gas should be performed to identify patients with carbon dioxide retention and to determine the peri-operative oxygen requirements. Patients with significant pulmonary dysfunction should be evaluated by a pulmonologist pre-operatively.

Wound infection, deep venous thrombosis and pulmonary embolism are all associated with obesity. Pre-operative antibiotic prophylaxis with cefazolin or vancomycin should be given at least 30 min before skin incision to allow for adequate tissue penetration.

To prevent a venous thromboembolic event, pneumatic compression devices should be placed on the calves pre-operatively. Place pneumatic compression stockings on the lower extremities of all obese parturients prior to and during surgery as prophylaxis against deep vein thrombosis, ensuring that the compression stockings remain in place until the patient is fully ambulatory. For short-out patient procedures, this is probably sufficient prophylaxis. For longer surgeries or surgeries performed under general anaesthesia, heparin prophylaxis is recommended. Most authors recommend unfractionated heparin 5,000 IU or low-molecular weight heparin every 12 h starting before surgery and continuing until the patient is ambulatory.

## CUFF SIZE FOR BLOOD PRESSURE

Another problem that the anaesthesiologist often encounters when dealing with morbidly obese patients is difficulty with non-invasive blood pressure monitoring. Unless the length of the cuff exceeds the circumference of the arm by 20%, systolic and diastolic blood pressure measurements may overestimate true maternal blood pressure. Direct arterial pressure measurement may be useful in the morbidly obese women where sphygmomanometry is often inaccurate, especially in patients with comorbidities such as chronic hypertension and pre-eclampsia. An intra-arterial catheter also offers the advantage of having the opportunity to perform repeated blood gas sampling, if indicated.

## OPERATING TABLE AND POSITION

### Operating table

Selection of appropriate operating table should occur before surgery. Standard operating tables can hold up to 450 lbs, but tables capable of holding up to 1,000 lbs are available and may be necessary for morbidly obese individuals. An appropriately sized operating table is imperative. The use of two operating tables (side by side) has been described.[27] The problem with this technique is that it is impossible to raise, lower or change the position of the tables in a completely synchronous manner. Another possibility is to use one set of arm boards, placed parallel to the operating table to extend the width of the table, while an extra set of arm boards can be used to position the arms of the patient.

### Position

**Figure 4:**
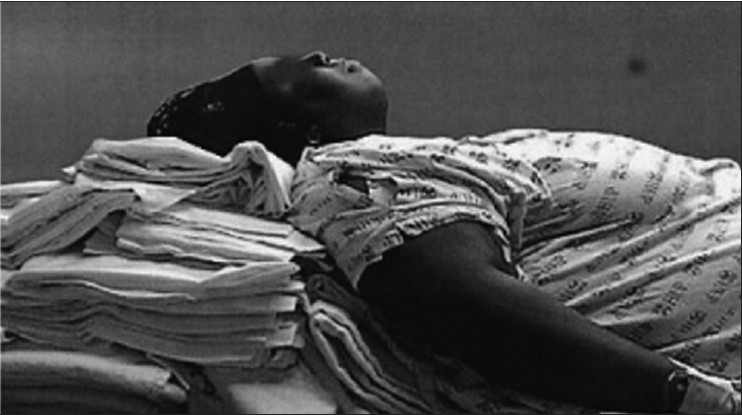
Positioning

All morbidly obese patients undergoing caesarean section should be placed in a ramped position with left uterine displacement regardless of primary anaesthetic technique. The ramped position has been shown to improve the laryngoscopic view. The effect may be even more important for parturients with a large breast, which can obstruct the insertion of the laryngoscopic blade. In the ramped position, blankets are folded under the chest and head to achieve the horizontal alignment with external auditory meatus and sternal notch [Figure 4].[28] This position aligns the oral, pharyngeal and tracheal access and frees the mandible to accommodate the tongue and the laryngoscopic blade. A 30° head-up position may minimize the impact on the respiratory mechanics and oxygenation.

Cephalad retraction of the heavy panniculus can cause aortocaval compression, maternal hypotension, non-reassuring foetal heart tones and, also, foetal death.[29]

## ANAESTHESIA

### Caesarean section regional techniques

#### Continuous lumbar epidural analgesia

A higher percentage of morbidly obese parturients will require cesarean delivery compared with non-obese parturients. Epidural anaestheisa offers several advantages. First, the ability to titrate the dose to achieve the desired level of analgesia, ability to extend the block for prolonged surgery, a decreased incidence and, perhaps, slow speed of developing hypotension and utilization for post-operative analgesia. In obese parturients, the administration of local anaesthetic should be closely titrated using a small incremental dose. Epidural anaesthesia alone is usually well tolerated in the obese parturients. The level of analgesia should be carefully tested before the surgeon is allowed to begin the procedure.

Extending labour analgesia for caesarean section requires additional local anaesthetic of higher concentration than the dilute solutions used to provide labour analgesia. The level of anaesthesia required for caesarean section is at least T4-5. Following an injection of the test dose, many anaesthesiologists administer incremental doses of 2% lidocaine with epinephrine until the desired effect is attained. Bupivacaine 0.5% can also be used.

Prophylactic placement of an epidural catheter when not contraindicated in labouring morbidly obese women would potentially decrease the anaesthetic and perinatal complications associated with attempts at emergency provision of regional or general anaesthesia.

## POSITION

The sitting position is more preferred because the line joining the occiput or the prominence of C7 and the gluteal cleft can be used to approximate the position of the midline sitting position, which allows the fat of the back to settle laterally and symmetrically and improves the identification of the midline. Morbidly obese woman tend to be more comfortable sitting on the side of the bed with a stool placed under their feet.

The horizontal lateral recumbent head-down position reduces the incidence of intravascular placement by reducing the venous congestion in the epidural veins.[30]

## NUMBER OF ATTEMPTS

Jordan and others noted that 74.4% of these patients needed more than one attempt for successful epidural needle placement.

## DURAL PUNCTURE

There is 4% incidence of dural puncture in morbidly obese parturients[31]

### Epidural space distance from skin

Hamza and others[32] found that the distance from the skin to the epidural space was significantly shorter when the epidural was performed with the patient in a sitting position as compared with the lateral decubitus position.[33] Computed tomography (CT) was used to measure the depth of the epidural space in non-pregnant patients. It is sufficient to use a standard epidural needle for the first attempt. BMI is a poor predictor of distance to the epidural space.[34]

## IDENTIFICATION OF MIDLINE

### Patient assists

**Figure 5:**
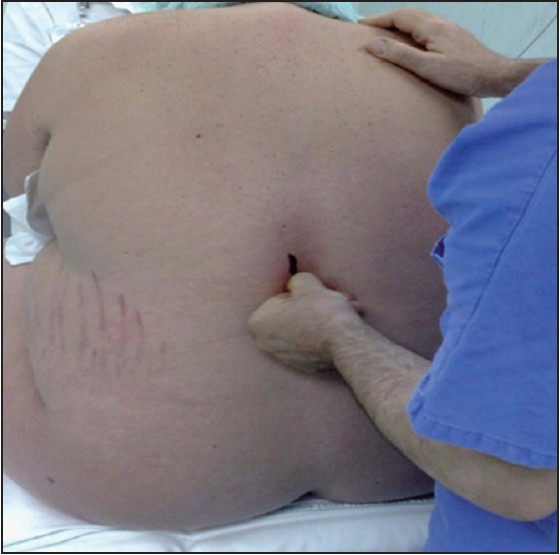
Identification of midline [Source: Expert Rev of Obstet Gynecol © 2009 Expert Reviews Ltd]

The parturient assists the anaesthesiologist verbally by indicating whether she feels the needle more on the left or on the right side of the spine,[35] which may prove to be a valuable tool when trying to identify the midline in these morbidly obese patients [Figure 5].

Needle help guide[36] uses an 8.5 cm 26 needle to probe for the posterior process of the lumbar vertebra. When the lumbar process is located, it can be used as a landmark for epidural needle insertion.

In case difficult epidural placement is encountered, ultrasound image should be considered.[37,38] Grau and others suggested that the quality of images obtained with the paramedian longitudinal approach is superior compared with images obtained with the transverse and median longitudinal approaches. The transverse approach is easier to perform. It is often difficult in obese patients to identify the shadow of the spinal process. Instead, the symmetry of the paraspinal muscles can be used.

## CORRECT PLACEMENT OF CATHETER AND FIXATION

The risk of epidural catheter dislodgement is increased in obese patients. Sliding of skin over the subcutaneous tissue has been proposed as an important factor in epidural catheter migration.[39] Iwama and Katayama[40] noticed a 3 cm skin movement in some patients. To avoid the tendency of epidural catheter to walk, the catheter is placed 7 cm in the epidural space. Hamilton and others[41] demonstrated that the epidural catheter not fixed at the skin could move 1–2.5 cm inward when the parturient posture is changed from the sitting to the lateral recumbent position, with the greatest change seen in patients with BMI >30. Suturing the epidural catheter to the skin using an adhesive dressing has been recommended.[42] Nevertheless, the failure rate of the epidural catheter in the general obstetric population varies between 8% and 13%,[43,44] with major causes of failure being no analgesia.

## COMBINED SPINAL EPIDURAL ANAESTHESIA

Combined spinal epidural anaesthesia (CSE) has become a well-established alternative to epidural analgesia. This provides a faster onset of effective pain relief and increases patient satisfaction. The potential drawback of CSE is that the location of the epidural catheter is initially uncertain. In an emergency, this unproven catheter may fail to provide adequate anaesthesia. On the other hand, studies[43,45–47] have shown that catheter inserted as part of the CSE technique produces anaesthesia more reliably than that via a standard epidural technique. The appearance of cerebrospinal fluid (CSF) at the hub of the spinal needle indirectly confirms the correct epidural needle placement. This increases the likelihood of a proper working catheter. Lower epidural analgesic requirements have been reported in obese parturients when compared with normal patients, probably secondary to a reduced volume in their epidural and subarchanoid space due to increased abdominal pressures.[48,49]

## SPINAL ANAESTHESIA

Single-shot spinal anaesthesia remains the most common type of anaesthesia employed for delivery of the foetus by caesarean section. The advantage of using subarachnoid block includes a dense reliable block of rapid onset. However, technical difficulties comprises of potential for high spinal blockade, profound dense thoracic motor blockade leading to cardiorespiratory compromise and inability to prolong the blockade. It is widely believed that local anaesthetic requirements are lower in pregnant patients and that the duration of surgery may extend beyond the duration of single-shot spinal anaesthsia. In such cases, intra-operative induction of general anaesthesia is undesirable and potentially hazardous.

## CONTINUOUS SPINAL ANALGESIA

With the unreliability of the epidural placement of the catheter, it is often preferred to conduct an intentional continuous spinal analgesia. Accidental dural puncture during epidural space identification can be converted as continuous spinal analgesia. This technique provides considerable predictability and reliability, allowing good control of the anaesthetic level and duration of block. The catheter is introduced 2–3 cm into the subarchanoid space. The low incidence of post-dural puncture headache may be attributed to the engorged extradural veins and the large amount of extradural fat, which reduce the CSF leak.[50] In a study, Michaloudis and others found that continuous spinal anaesthesia was useful for the peri-operative management of morbidly obese patients undergoing laparotomy for gastroplastic surgery.

## GENERAL ANAESTHESIA CONSIDERATIONS

General anaesthesia imposes great discipline and plan on the part of the anaestheisologist in balancing the altered physiology and anatomy and, applying the pharmacological knowledge on a huge mass of fat, the anatomical and physiological changes caused by both obesity and pregnancy are less favorable to anaesthetists, resulting in an increased incidence of difficult intubation and rapid desaturation during the apnoeic phase.

## AIRWAY ISSUES

A “difficult airway” has been defined as the clinical situation in which a conventionally trained anaesthesiologist experiences problems with mask ventilation, with tracheal intubation or with both.[51] The tracheas of obese patients are believed to be more difficult to intubate than those of normal weight patients.[52–54]

### Equipment for difficult intubation

#### Mayo clinic

1. Flexible fiberoptic bronchoscope
2. Bullard laryngoscope, Circon, Stanford, CT, USA
3. ProSeal laryngeal mask airway, LMA North America, San Diego, CA, USA
4. Intubating laryngeal mask airway
5. Combitube, Kendall-Sheridan Catheter, Argyle, MA, USA
6. Trachlight, Laedal Medical, New York, NY, USA
7. Jet ventilation apparatus
8. Cricothyrotomy Seldinger kit

Difficult intubation is defined as inadequate exposure of the glottis by direct laryngoscopy.

Voyagis and others reported that difficult intubation increases with increasing BMI.[53] Factors that have been associated with difficult laryngoscopy include short sternomental distance, short thyromental distance, large neck circumference, limited head, neck and jaw movement, receding mandible and prominent teeth.[55,56] Of these factors, only large neck circumference was associated with problematic intubation.[57] Logistic regression identified neck circumference as the best single predictor of problematic intubation. Neck circumference was measured at the level of the superior border of the cricothyroid cartilage. Problematic intubation was associated with increasing neck circumference and a Mallampati score of 3.

## AIRWAY ASSESSMENT

Most airway catastrophes occur when airway difficulty is not recognized before induction of anaesthesia. Timely evaluation of the parturient’s airway and adequate preparation to deal with the airway in the non-emergent setting are helpful in avoiding airway catastrophes.

There are a few simple pre-operative bedside determinations that can be performed quickly to evaluate the airway in a pregnant patient. These include, but are not limited to, mouth opening, Mallampati class,[58,59] thyromental distance and atlanto occipital extension. *It is recommended that the airway be reassessed before induction of general anaesthesia*.[60]

The ability to protrude the mandible should be assessed. The ability of the lower incisors to protruded anterior to the upper incisors rarely poses difficulty in intubation.[61]

## CAESAREAN SECTION IN ANTICIPATED DIFFICULT AIRWAY SITUATION

When a caesarean section has to be performed in an anticipated difficult situation, we are left with three options: awake intubation, regional anaesthesia and local anaesthesia.

## AWAKE FIBEROPTIC INTUBATION

Full-aspiration prophylaxis should be instituted before intubation. An anticholinergic drying agent such as glycopyrrolate allows better application and absorption of local anaesthetics to the airway mucosa and thus improves visualization of the oropharyngeal structures. The route of fiberscopic intubation is important in pregnant patients. The nasal mucosa is engorged in pregnancy and, despite vasoconstriction, this can precipitate epistaxis, leading to a compromised airway. The oral route is commonly used and preferred. Topical anaesthesia is the primary anaesthetic for an awake intubation. It can be achieved with a spray of lidocaine at the base of the tongue and lateral pharyngeal walls along with application of lidocaine jelly to the base of the tongue via a tongue blade. Sufficient time must be allowed to anaesthetize all portions of the airway. This helps to minimize the swallowing and gag reflexes. The larynx and trachea can be topically anaesthetized by injection of lidocaine through the cricothyroid membrane or via the suction port of the fiberscope.[62] The patient is at risk for aspiration if regurgitation or vomiting takes place after topical anaesthesia and before the airway is secured. A shorter interval between application of topical anaesthesia and tracheal intubation lessens the potential of aspiration.[63]

## REGIONAL ANAESTHESIA

Regional anaesthesia is the best possible choice in most cases of anticipated difficult airway. Either spinal or epidural anaesthesia is acceptable, provided no contraindications exist in the absence of foetal compromise. When a caesarean section is non-emergent, epidural anaesthesia can be used. When time is limited, spinal anaesthesia is the choice. The advantages of regional anaesthesia include the following: the mother is awake and can protect her airway, airway manipulation is not necessary; the incidence of acid aspiration is decreased. If regional anaesthesia is administered to a patient with difficult airway, close monitoring by an experienced anaesthesiologist is essential.

## LOCAL ANAESTHESIA

In the developing countries, this method is still used when the emergency condition of the parturient demands immediate intervention. In India, where there are certain communities with pseudocholenesterase deficiency, this poses a special problem. In those situations, succinyl choline is not given. Then, the anaesthesiologist is left with firbreoptic intubation or local anaesthesia. The awake mother has a protective airway.

## CAESAREAN SECTION: UNANTICIPATED DIFFICULT AIRWAY

In a patient requiring an emergency caesarean section for foetal distress and failed intubation, management goals include maternal oxygenation, airway protection and prompt delivery of the baby. If possible, consider returning to spontaneous ventilation, awakening the mother and calling for help.

### Failed initial attempts at intubation

The recommendation in the case of a grade III laryngoscopic view is that no more than three attempts at laryngoscopy and intubation should be made. In a grade IV laryngoscopic view, the Difficult Airway Algorithm should be followed without delay.[64] Call for help immediately if surgery needs to be performed.

### Non-emergent pathway: Can ventilate, cannot intubate situation

In an elective caesarean where we can ventilate but cannot intubate, mask ventilation is continued with cricoid pressure until the patient is fully able to protect her airway. Adequate oxygenation without aspiration is the goal.

## LARYNGEAL MASK AIRWAY

As per practice guidelines 2003 for difficult airway, Laryngeal mask airway (LMA) is the tool of choice in a can ventilate, cannot intubate (CVCI) situation. LMA has revolutionized management of difficult airway. LMA should be used earlier rather than later following failed endotracheal intubation. Han and colleagues reported the successful use of LMA as a ventillatory device in 1,060 of 1,067 patients for elective caesarean delivery.[65] In a German survey, LMAs were available in 91% of the obstetrics departments, similar to figures from the United Kingdom (91.4%). According to the same survey, 72% of the anaesthesiologists favoured LMA as the first treatment option for the CVCI situation.[66] In a survey in the United Kingdom, 71.8% of the obstetrical anaesthesiologists advocated use of LMA in a CVCI situation. Eight anaesthesiologists stated that LMA proved to be a “lifesaver”.[67] Recently, 18 obstetrics units in Ireland were surveyed for difficult airway equipment. All of the units had LMA as an alternative device for ventilation and intubation. Fifty percent of the units also had an intubating laryngeal mask airway (ILMA) among their airway equipment.[68] zri and colleagues conducted a survey in Israel to evaluate the practices of Israeli anaesthetists regarding familiarity with airway devices. Ninety-six percent of the anaesthetists were skilled with LMAs and 73% with fiberoptics. Of the obstetrical rooms surveyed in this study, only 36% were equipped with laryngeal masks, 24% with fiberscopes and 22% with equipment for tracheal puncture.[69]

## PROSEAL-LMA

The design of the proseal-lma (PLMA) reliably allows positive pressure ventilation up to 30–40 cm H_2_O. Thus, the seal is 10 cm H_2_O higher, giving it greater ventillatory capability than the classic LMA. The PLMA has been successfully used in parturients after failed intubation during rapid-sequence induction.[63,70,71]

## ILMA

ILMA has also been used in parturients after failed intubation.[72,73]

## LARYNGEAL TUBE

Laryngeal tube (LT) is a new supraglottic airway device. LT is a newer generation LT that is fitted with a second lumen for suctioning and gastric drainage. LTs has been recently used in a parturient having an urgent cesarean section in a CVCI situation.[72,74]

## COMBITUBE

Combitube has been successfully used for the management of failed intubation in caesarean delivery.[75] Combitube provides as option for blind intubation of either the oesophagus or the trachea. In either position, the patient can be oxygenated and ventilated and the airway is protected against aspiration of gastric contents. Combitube is successfully used for the management of failed intubation in caesarean delivery.[75]

### Transtracheal jet ventillation

It is probably the fastest route to oxygenation in a desaturating patient.

### Cricothyroidotomy and surgical tracheostomy

Percutaneous cricothyrotomy is safe, quick and easy to perform as Transtracheal jet ventillation (TTJV).[76]

## FAILED INTUBATION DRILL

**Figure 6:**
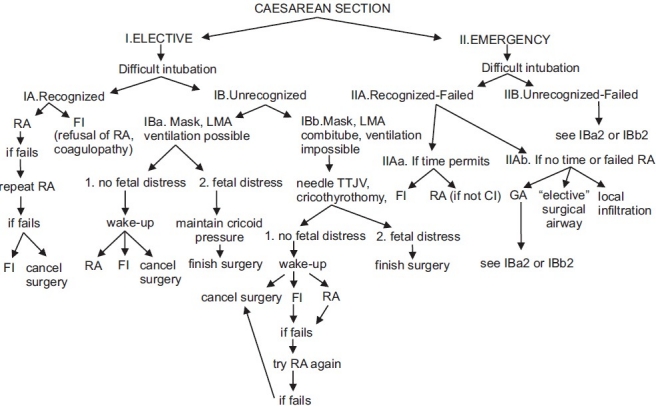
Difficult airway algorithm

If the initial attempts to intubate the trachea fail, it is critical to follow a difficult air way algorithm [Figure 6]. Focus on maternal oxygenation mask ventilation is best achieved with an oral airway and three people, one to apply cricoid pressure, a second to maximize jaw thrust and a third to squeeze the bag and monitor the patient. If ventilation fails, the team should insert a supraglottic air way device and prepare to create a surgical airway. The LMA is the preferred choice by many anaesthesiologists. In elective cases, fiberoptic intubation is considered.

## GENERAL ANAESTHESIA PROCEDURE

### General anaesthesia considerations: Prevention of acid aspiration and its related precautions

It is standard practice to administer 30 ml of non-particulate antacid 0.3 M sodium citrate 30 min before the initiation of any anaesthetic being administered to the patient. H_2_ antagonist, such as ranitidine or a proton pump inhibitor, such as omeprazole, the evening before and again 60–90 min before the induction of anaesthesia further reduce gastric acidity, and volume prokinetic agents like inj metaclopromide may help further, especially in diabetes-associated patients.[77–80]

## PRE-OPERATIVE OXYGENATION

Pre-oxygenation and denitrogenation is crucial in these patients before induction of general anaesthesia. The most common method is 3–5 min of 100% oxygen breathing. Baraka *et al*.[81] showed that pre-oxygenation achieved by eight deep breaths within 60 s at an oxygen flow of 10 L/min not only resulted in a higher PaO_2_ but also in a slower haemoglobin desaturation compared with the four deep breathes technique.

## INDUCTION AND MAINTENANCE

Induction may be achieved with pentothal sodium 4 mg/kg and up to 500 mg can be done as per the unit body weight. Prolonged duration of action is expected due to increased central volume distribution and prolonged elimination half-life. Intubation can be achieved with succinyl choline 1–1.5 mg/kg up to 200 mg. Plasma cholinesterase activity is increased in the obese requiring an initial larger dose. Capnography and bilateral lung auscultation should be used to confirm successful intubation before surgical incision. Patients with morbid obesity experience further decrease in FRC under general anaesthesia. Techniques to maintain oxygenation include (1) increase tidal volume to 12—15 ml/kg, (2) increase FIO_2_ >50%, (3) head up and (4) panniculus suspension.

Isoflurane, sevoflurane and desflurane are all used in standard concentrations in obese parturients. Desflurane allows faster recovery when compared with sevoflurane. Dense intra-operative neuromuscular blockade is best achieved by titrating intermediate-acting agents using a twitch monitor. Emergence, extubation and recovery represent critical periods for obese woman who deliver under general anaesthesia. (1) To maximize the safety during this period, ensure adequate return of muscle function with a nerve stimulator and neostigmine reversal, (2) insert an orogastric tube to empty the stomach just before emergence, (3) delay extubation until the patient is completely awake and is able to meet the intensive care extubation criteria, (4) administer oxygen and (5) continue monitoring.

## POST-OPERATIVE CARE

Obese parturients are at increased risk of post-operative complications such as hypoxaemia, atelectasis and pneumonia, deep vein thrombosis and pulmonary embolism, pulmonary oedema, post-partum cardiomyopathy, post-operative endometritis and wound complications such as infection and dehiscence.[82,83] Early mobilization, thromboprophylaxis, aggressive chest physiotherapy and adequate pain control are the key to the success of effective post-operative care. Nursing in the reclined position and oxygen supplementation can potentially reduce critical respiratory events.

Early mobilization has been shown to improve the respiratory volumes in the immediate post-operative phase.[84] Interestingly, Hood and Dewan found that, in morbidly obese women, all post-partum complications occurred in those undergoing caesarean section and not in those having vaginal delivery.[83] Pain control should be adequate in the post-operative period to facilitate mobilization and chest physiotherapy as it is one of the determinants of post-operative maternal morbidity. Epidural analgesia has been shown to improve the post-operative respiratory function in patients undergoing abdominal surgery.[85] Epidural infusion of local anaesthetic with opioids improves the quality of dynamic post-operative pain relief.[86] Patient-controlled intravenous opioids have also been successfully used for post-operative pain relief in the morbidly obese.[87] Thromboembolic episodes remain the leading cause of direct maternal deaths in the UK. Obesity is a known independent risk factor for deep vein thrombosis. Both pharmacological and mechanical strategies are used for thromboprophylaxis, and an adequate dose of an anticoagulant for an appropriate duration is recommended. Obesity cardiomyopathy is a well-recognized clinical entity and at least three cases of peripartum cardiomyopathy in obese patients have been reported.[83,88,89] Wound complications occur more frequently in obese than in non-obese patients and often lead to prolonged recovery. They have been found to be increased with midline abdominal incision compared with Pfannenstiel incision.[90] Hospital stay and costs have been found to be increased for morbidly obese patients after both vaginal delivery and caesarean section.[91]

## GUIDELINES RECOMMENDED

All obstetric units should develop protocols for the management of morbidly obese women. These should include pre-assessment procedures, special community, ward and theatre equipment such as large sphygmomanometer cuffs, hoists, beds and operating tables and long regional block needles Morbidly obese women should be referred for anaesthetic assessment and advice as part of their antenatal care management by consultant anaesthetists is essential and difficulties with airway management and intubation should be anticipated. Positioning the women requires skill and sufficient manpower in the event of a requirement for induction of general anaesthesia is essential.[92] Direct arterial pressure measurement may be useful in the morbidly obese women where sphygmomanometry is often inaccurate. All morbidly obese women in childbirth should be given prophylactic low-molecular weight heparin, and the duration of therapy needs to be determined in view of likely immobility. Thromboembolic stockings of an appropriate size need to be available.

## FAT IS NOT A THREE-LETTERED WORD. IT KILLS THE WORLD

### What is new obstetric anaesthesia?

1. Ephedrine versus phenylephrine to treat hypotension after spinal anaesthesia: investigators compared varying combinations of the two drugs given by infusion to keep the blood pressure at baseline. Haemodynamic control was better for the mother and acid–base status was better in the foetus when phenylephrine was used instead of ephedrine.
2. Low-dose bupivacaine with phenylephrine provided the best haemodynamic stability during subarachnoid block.
3. Mallampati classification is not static and should be assessed just before instrumentation. Mallampati score ≥3 and large neck circumferences were most useful and it is suggested that neck circumference should be included in our pre-operative assessment.
4. Oxytocin bolus produced hypotension, tachycardia, chest pain and signs of myocardial ischaemia on 12-lead ECG. Oxytocin is not safe to give as an IV bolus.
5. Embolism remains the #1 cause of maternal death in the US.
6. Spinal anaesthesia is preferred in severe pre-eclampsia.

## References

[1] CIO Foundation. General Overweight and Obesity Statistics.

[2] Sellmann S (2010). An effective solution to the obesity epidemic Nexus Mag.

[3] (2005). American College of Obstetricians and Gynecologists. ACOC Committee Opinion number 315, Obesity in pregnancy. Obstet Gynecol.

[4] Ogden CL, Carroll MD, Curtin LR, McDowell MA, Tabak CJ, Flegal KM Prevalence of overweight and obesity in the United states, 1999-2004.

[5] Ogden CL, Cerrol MD (2006). Prevelance of overweight and obesity in united states 1999-2004. JAMA.

[6] Knight M, Kurinczuk JJ, Spark P, Brocklehurst P (2010). UK Obstetric Surveillance System. Extreme obesity in pregnancy in the United Kingdom. Obstet Gynecol.

[7] (2007). India facing obesity epidemic: experts.

[8] Khokhar KK, Kaur G, Sidhu S (2010). Prevalence of obesity in working premenopausal and postmenopausal women of Jalandhar district, Punjab. J Hum Ecol.

[9] Das M, Bose K (2006). Presence of high rates of overweight and obesity among adult Marwaris of Howrah, West Bengal, India. Coll Antropol.

[10] Gupta R, Sarna M, Thanvi J, Rastogi P, Kaul V, Gupta VP (2004). High prevalence of multiple coronary risk factors in Punjabi Bhatia community: Jaipur Heart Watch-3. Indian Heart J.

[11] Saha UC, Saha KB (2010). A trend in women’s health in India - what has been achieved and what can be done. Rural Remote Health.

[12] Regional Medical Research Centre for Tribals (ICMR), Jabalpur, MP, India. http://www.rmrct.org.

[13] (2006). International Institute for Population Sciences. National Fact Sheet. India National Family Health Survey (NFHS)-3, 2005-6.

[14] Third National Family Health Survey. International Institute for Population Sciences, Mumbai.

[15] Wong CA, Loffredi M, Ganchiff JN, Zhao J, Wang Z, Avram MJ (2002). Gastric emptying of water in term pregnancy. Anaesthesiology.

[16] Macfie AG, Magides AD, Richmond MN, Reilly CS (1991). Gastric emptying in pregnancy. Br J Anaesth.

[17] Sandhar BK, Elliott RH, Windram I, Rowbotham DJ (1992). Peripartum changes in gastric emptying. Anesthesia.

[18] Abrams BF, Laros RK (1988). Overweight and pregnancy complications. Int J Obes.

[19] Weiss JL, Malone FD, Emig D, Ball RH, Nyberg DA, Comstock CH (2004). Obesity, obstetric complications and cesarean delivery rate—a population- based screening study. Am J Obstetric Gynecol.

[20] Cooper GM, McClure JH (2008). Anaesthesia chapter from Saving mothers’ lives; reviewing maternal deaths to make pregnancy safer. Br J Anaesth.

[21] Cedergren MI (2004). Maternal morbid obesity and the risk of adverse pregnancy outcome. Obstet Gynecol.

[22] Jolly MC, Sebire NJ, Harris JP, Regan L, Robinson S (2003). Risk factors for macrosomia and its clinical consequences: a study of 350,311 pregnancies. Eur J Obstet Gynecol Reprod Biol.

[23] Ehrenberg HM, Mercer BM, Catalano PM (2004). The influence of obesity and diabetes on the prevalence of macrosomia. Am J Obstet Gynecol.

[24] Edwards LE, Hellerstedt WL, Alton IR, Story M, Himes JH (1996). Pregnancy complications and birth outcomes in obese and normal-weight women: effects of gestational weight change. Obstet Gynecol.

[25] Nelson-Piercy C (2004). Thromboprophylaxis during pregnancy, labour and after vaginal delivery. RCOG Guideline No. 37.

[26] Lewis G (2007). The confidential enquiry into maternal and child health (CEMACH).Saving mothers’ lives: reviewing maternal deaths to make motherhood safer 2003-2005. The Seventh Report on Confidential Enquiries into Maternal Deaths in the United Kingdom.

[27] Gross TL (1983). Operative considerations in the obese pregnant patient. Clin Perinatol.

[28] Brodsky JB, Lemmens HJ, Brock-Utne JG, Saidman LJ, Levitan R (2003). Anaesthetic considerations for bariatric surgery. Proper positioning is important for laryngoscopy. Anaesth Analg.

[29] Hodgkinson R, Husain FJ (1980). Cesarean section associated with gross obesity. Br J Anaesth.

[30] Bahar M, Chanimov M, Cohen ML, Friedland M, Shul I, Gofman V (2004). The horizontal lateral recumbent head-down position reduces the incidence of epidural venous puncture during catheter insertion in obese parturisnts. Can J Anaesth.

[31] Faure E, Moreno R, Thisted R (1994). Incidence of postdural puncture headache in morbidly obese parturients. Reg Anesth.

[32] Hamza J, Smida M, Benhamou D, Cohen SE (1995). Parturient’s posture during epidural puncture affects the distance from skin to epidural space. J Clin Anesth.

[33] Bahk JH, Kim JH, Lee JS, Lee SC (1998). Computed tomography study of the lumbar (L3-4) epidural depth and its relationship to physical measurements in young adult men. Reg Anesth Pain Med.

[34] Watts RW (1993). The influence of obesity on the relationship between body mass index and the distance to the epidural space from the skin. Anaesth Intens Care.

[35] Wills JS, Bowie R, Bogod DG (2002). A pilot study of patient-led identification of the midline of the lumbar spine. Anaesthesia.

[36] Maitra AM, Palmer SK, Bachhuber SR, Abram SE (1979). Continuous epidural analgesia for cesarean section in a patient with morbid obesity. Anesth Analg.

[37] Grau T, Leipold RW, Horter J, Conradi R, Martin E, Motsch J (2001). The lumbar epidural space in pregnancy: visualization by ultrasonography. Br J Anaesth.

[38] Grau T, Leipold RW, Horter J, Conradi R, Martin EO, Motsch J (2001). Paramedian access to the epidural space: the optimum window for ultrasound imaging. J Clin Anesth.

[39] Faheem M, Sarwar N (2002). Sliding of the skin over subcutaneous tissue is another important factor in epidural catheter migration. Can J Anesth.

[40] Iwama H, Katayama T (1999). Back skin movement also causes ‘walking’ epidural catheter. J Clin Anesth.

[41] Hamilton CL, Riley ET, Cohen SE (1997). Changes in the position of epidural catheters associated with patient movement. Anesthesiology.

[42] Poulton B, Young P (2000). A novel method for epidural catheter fixation. Anaesthesia.

[43] Eappen S, Blinn A, Segal S (1998). Incidence of epidural catheter replacement in parturients: a retrospective chart review. Int J Obstet Anesth.

[44] Riley ET, Papasin J (2002). Epidural catheter function during labour predicts anesthetic efficacy for subsequent cesarean delivery. Int J Obst Anes.

[45] van de Velde M, Teunkens A, Hanssens M, van Assche FA, Vandermeersch E (2001). Post dural puncture headache following combined spinal epidural or epidural anesthesia in obstetric patients. Anaesth Intensive Care.

[46] Pan PH, Bogard TD, Owen MD (2004). Incidence and characteristics of failures in obstetric neuraxial analgesia and anesthesia: a retrospective analysis of 19,259 deliveries. Int J Obstet Anesth.

[47] Norris MC (2000). Are combined spinal-epidural catheters reliable?. Int J Obstet Anesth.

[48] Hogan QH, Prost R, Kulier A, Taylor ML, Liu S, Mark L (1996). Magnetic resonance imaging of cerebrospinal fluid volume and the influence of body habitus and abdominal pressure. Anesthesiology.

[49] Panni MK, Columb MO (2006). Obese parturients have lower epidural local anaesthetic requirements for analgesia in labour. Br J Anaesth.

[50] Coker LL (2002). CRNA, MSN; continuous spinal anaesthesia for cesarean section for a morbidly obese parturient patient – A Case Report. AANA J.

[51] (1993). Practice guidelines for management of the difficult airway: a report by the American Society of Anesthesiologists Task Force on Management of the Difficult Airway. Anesthesiology.

[52] Fisher A, Waterhouse TD, Adams AP (1975). Obesity: its relation to anaesthesia. Anaesthesia.

[53] Voyagis GS, Kyriakis KP, Dimitriou V, Vrettou I (1998). Value of oropharyngeal Mallampati classification in predicting difficult laryngoscopy among obese patients. Eur J Anaesthesiol.

[54] Benumof JL (2001). Obstructive sleep apnoea in the adult obese patient: implications for airway management. J Clin Anesth.

[55] Wilson ME, Spiegelhalter D, Robertson JA, Lesser P (1988). Predicting difficult intubation. Br J Anaesth.

[56] Naguib M, Malabarey T, AlSatli RA, Al Damegh S, Samarkandi AH (1999). Predictive models for difficult laryngoscopy and intubation: a clinical, radiologic and three-dimensional computer imaging study. Can J Anaesth.

[57] Brodsky JB, Lemmens HJ, Brock-Utne JG, Vierra M, Saidman LJ (2002). Morbid obesity and tracheal intubation. Anesth Analg.

[58] Samsoon GL, Young JR (1987). Difficult tracheal intubation: A retrospective study. Anaesthesia.

[59] Cormack RS, Lehane J (1984). Difficult tracheal intubation in obstetrics. Anaesthesia.

[60] Farcon EL, Kim MH, Marx GF (1994). Changing Mallampati score during labour. Can J Anaesth.

[61] Rocke DA, Murray WB, Rout CC, Gouws E (1992). Relative risk analysis of factors associated with difficult intubation in obstetric anesthesia. Anesthesiology.

[62] Sanchez AF, Morrison DE, Hagberg CA (2000). Preparation of the patient for awake intubation. Handbook of Difficult Airway Management.

[63] Ovassapian A, Tuncbilek M, Weitzel EK, Joshi CW (2005). Airway management in adult patients with deep neck infections: A case series and review of the literature. Anesth Analg.

[64] Suresh MS (2001). Difficult airway in the parturient. Probl Anesthesia.

[65] Han TH, Brimacombe J, Lee EJ, Yang HS (2001). The laryngeal mask airway is effective (and probably safe) in selected healthy parturients for elective cesarean section: A prospective study of 1067 cases. Can J Anaesth.

[66] Stamer UM, Messerschmidt A, Wulf H, Hoeft A (2000). Equipment for the difficult airway in obstetric units in Germany. J Clin Anesth.

[67] Gataure PS, Hughes JA (1995). The laryngeal mask airway in obstetrical anaesthesia. Can J Anaesth.

[68] Niazi A, Cummins E, Walsh K (2004). Difficult airway equipment in obstetric units in the Republic of Ireland: Results of a national survey. Eur J Anaesthesiol.

[69] Keller C, Brimacombe J, Lirk P, Pühringer F (2004). Failed obstetric tracheal intubation and postoperative respiratory support with the ProSeal laryngeal mask airway. Anesth Analg.

[70] Awan R, Nolan JP, Cook TM (2004). Use of a ProSeal laryngeal mask airway for airway maintenance during emergency caesarean section after failed tracheal intubation. Br J Anaesth.

[71] Vaida SJ, Gaitini LA (2004). Another case of use of the ProSeal laryngeal mask airway in a difficult obstetric airway. Br J Anaesth.

[72] Minville V, N’guyen L, Coustet B, Fourcade O, Samii K (2004). Difficult airway in obstetrics using Ilma-Fastrach. Anesth Analg.

[73] González González G, Marenco de la Fuente ML, Bertomeu Cornejo M (2005). Fastrach mask to resolve a difficult airway during emergency cesarean section [in Spanish]. Rev Esp Anestesiol Reanim.

[74] Zand F, Amini A (2005). Use of the laryngeal tube-S for airway management and prevention of aspiration after a failed tracheal intubation in a parturient. Anesthesiology.

[75] Wissler RN (1993). The esophageal-tracheal Combitube. Anesthesiol Rev.

[76] Munnur U, de Boisblanc B, Suresh MS (2005). Airway problems in pregnany. Crit Care Med.

[77] O’Sullivan GM, Bullingham RE (1984). The assessment of gastric acidity and antacid effect in pregnant women by a noninvasive radiotelemetry technique. Br J Obstet Gynaecol.

[78] O’Sullivan GM, Guyton TS, Chestnut DH (2004). Aspiration: risk, prophylaxis, and treatment. Obstetric anesthesia.

[79] Orr DA, Bill KM, Gillon KR, Wilson CM, Fogarty DJ, Moore J (1993). Effects of omeprazole, with and without metoclopramide, in elective obstetric anaesthesia. Anaesthesia.

[80] Stuart JC, Kan AF, Rowbottom SJ, Yau G, Gin T (1996). Acid aspiration prophylaxis for emergency caesarean section. Anaesthesia.

[81] Baraka AS, Taha SK, Aouad MT, El-Khatib MF, Kawkabani NI (1999). Preoxygenation comparision of maximal breath in and tidal volume breathing techniques. Anaesthesiology.

[82] Perlow JH, Morgan MA (1994). Massive maternal obesity and perioperative cesarean morbidity. Am J Obstet Gynecol.

[83] Hood DD Dewan DM (1993). Anesthetic and obstetric outcome in morbidly obese parturients. Anesthesiology.

[84] Von Ungern-Sternberg BS, Regli A, Bucher E, Reber A, Schneider MC (2004). Impact of spinal anaesthesia and obesity on maternal respiratory function during elective caesarean section. Anaesthesia.

[85] Ballantyne JC, Carr DB, deFerranti S, Suarez T, Lau J, Chalmers TC (1998). The comparative effects of postoperative analgesic therapies on pulmonary outcome: cumulative meta-analyses of randomised, controlled trials. Anesth Analg.

[86] Wheatley RG, Schug SA, Watson D (2001). Safety and efficacy of postoperative epidural analgesia. Br J Anaesth.

[87] Levin A, Klein S, Brolin R, Pitchford DE (1992). Patient controlled analgesia for morbidly obese patient: An effective modality if used correctly. Anesthesiology.

[88] Kaufman I, Bondy R, Benjamin A (2003). Peripartum cardiomyopathy and thromboembolism; anesthetic management and clinical course of an obese, diabetic patient. Can J Anaesth.

[89] Shnaider R, Ezri T, Szmuk P, Larson S, Warters RD, Katz J (2001). Combined spinalepidural anesthesia for cesarean section in a patient with peripartum dilated cardiomyopathy. Can J Anaesth.

[90] Wall PD, Deucy EE, Glantz JC, Pressman EK (2003). Vertical skin incisions and wound complications in the obese parturient. Obstet Gynecol.

[91] Galtier-Dereure F, Montpeyroux F, Boulot P, Bringer J, Jaffiol C (1995). Weight excess before pregnancy: complications and cost. Int J Obes Relat Metab Disord.

[92] Saravanakumar K, Rao SG, Cooper GM (2006). Obesity and obstetric anaesthesia. Anaesthesia Anaesthesia.

